# MicroRNA-in drug resistance

**DOI:** 10.18632/oncoscience.2

**Published:** 2014-01-13

**Authors:** Haoran Li, Burton B Yang

**Affiliations:** Sunnybrook Research Institute, Sunnybrook Health Sciences Centre, Toronto, and Department of Laboratory Medicine and Pathobiology, University of Toronto, Toronto

Colorectal cancer (CRC) is one of the leading causes of cancer mortality worldwide. Over the last three decades, considerable progress has been made on the treatment of CRC. For example, it has been estimated that over 80% of patients diagnosed with CRC after 2010 received chemotherapy [1]. Despite considerable success however, resistance to chemotherapeutic treatment has emerged as an obstacle to effective treatment. Thus, there is a growing need in identifying critical molecular biomarkers for predicting both the clinical outcome of chemotherapy as well as patients at risk of developing drug resistance. Recently, it has become clear that multiple drug resistance (MDR) arises as a consequence of an accumulation of genetic and epigenetic alternations. Among them, the microRNA (miRNA) network has been identified as a master regulator of MDR.

Currently, several laboratories are exploring how microRNAs manipulate drug resistance to cause CRC tumor relapse through epigenetic modulations. Recent research from our lab, published in Oncotarget (Fang et al., Oncotarget, 2014, Advance Publications) [2], uncovered the underlying mechanism responsible for acquired drug resistance and distant metastasis in CRC patients. Tumor samples of patients undergoing neoadjuvant chemotherapy were collected and microarrays were conducted by an independent group. Corresponding clinical outcomes were recorded according to the Response Evaluation Criteria in Solid Tumors (RECIST 1.1), and samples they were categorized into either chemosensitive or chemoresistant groups. By comparing miRNA expression patterns between these two groups, it was shown that microRNA-17 (miR-17) was consistently elevated in the chemoresistant group. We hypothesized that miR-17 might be a predictive factor of chemotherapeutic response in colorectal cancer. We found that high levels of miR-17 expression was closely correlated with worsened long-term survival in 81 patients receiving chemotherapy (5.26 vs. 7.29 yrs). To confirm the role of miR-17 in inducing MDR, we stably transfected the CRC cell lines, COLO205 and SW480 with a miR-17 overexpression plasmid. Indeed, MDR was correlated with miR-17 expression in a dose-dependent manner. Less drug-induced apoptosis was noted in CRC cells which highly expressed miR-17. In addition, knocking down miR-17 by antisense oligo was found to sensitize cells to cytotoxic agents' treatment.

It has long been thought that resistance to chemotherapy can be divided into two categories: innate and acquired [3]. Now this theory is facing challenges with the emergence of new evidence. In this paper, we reported that the levels of miR-17 were dramatically increased under chemotherapeutic agents' treatment. We believe that such a change confers an increased capacity for tumor cells to survive in stressed condition. Thus miR-17 over-expressing tumor cells have an innate survival advantage. Following the selective pressure of chemotherapy, this eventually leads to the accumulation of miR-17 expressing tumor cells in the surviving fraction. We found that high expression of miR-17 completely knocked down its target tumor suppressor PTEN, leading to excessive activation of downstream components such as AKT and HIF-1α. HIF-1α is capable of promoting miR-17 transcription, which in turn increases miR-17 accumulation [4]. Given this positive feedback loop, miR-17 concentration is continually increased after drug treatment, leading to a subsequent decrease in PTEN expression. These findings, in line with clinical data, provided direct insight into how miR-17 confers a poor prognosis by affecting tumor sensitivity to chemotherapy. Adding another layer of complexity however, single microRNAs can also act pleiotropically to target several pathways. Our understanding of these microRNA networks will help us bridge the link between innate and acquired drug resistance, and illuminate how this transition can occur at the post-transcriptional level.

There is now growing evidence that miR-17 plays a key role in determining the prognosis of colorectal cancer. Yu and colleagues reported that high miR-17 expression was associated with reduced overall survival in 96 colon cancer patients [5]. These findings were in agreement with results published by Ma et al [6]. Transfecting miR-17 oligo mimics into LoVo cells, the authors showed that miR-17 regulated CRC tumorigenesis by targeting P130. This study was an important contribution to our understanding of miRNAs in colorectal cancer. To further reveal the crosstalk between the microRNA network and tumor microenvironment, we generated stably transfected cell lines and showed temporal changes in miR-17 expression in CRC cells exposed to chemotherapy. With extensive investigation, PTEN/PI3K/AKT/HIF-1alpha cascade has been shown as one of the most crucial pathways which are responsible for therapy response and oxidative stress [7]. Therefore, it is with great value to decipher how epigenetic alterations of PTEN pathway can result in chemotherapeutic drug resistance.

**Figure:**
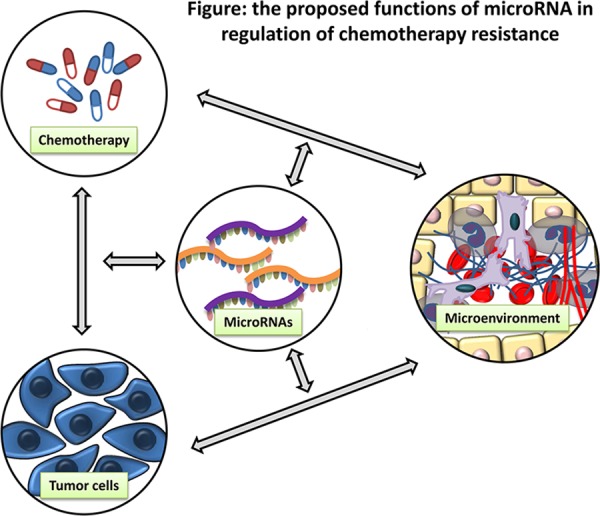
the proposed functions of microRNA in regulation of chemotherapy resistance

Given its inherent genomic instability, CRC is able to maintain growth and proliferation through cross-talk between several signaling pathways. This level of complexity makes it more challenging to successfully treat patients. In recognition of the important role of microRNAs in drug resistance, future research should be focused on therapeutic strategies that target this dysfunctional network.
